# Prediction of non-responders to oral appliance treatment of obstructive sleep apnea: a pilot study

**DOI:** 10.1007/s11325-025-03315-1

**Published:** 2025-04-15

**Authors:** Ulrik Leidland Opsahl, Morten Berge, Sverre Lehmann, Bjørn Bjorvatn, Anders Johansson

**Affiliations:** 1 Department of Clinical Dentistry, Faculty of Medicine, Center for Translational Oral Research (TOR), University of Bergen, Bergen, Norway; 2 Norwegian Competence Center for Sleep Disorders, Haukeland University Hospital, Bergen, Norway; 3 Department of Thoracic Medicine, Haukeland University Hospital, Bergen, Norway; 4 Department of Global Public Health and Primary Care, University of Bergen, Bergen, Norway; 5 Department of Clinical Science, University of Bergen, Bergen, Norway; 6 Department of Clinical Dentistry – Prosthodontics, Faculty of Medicine, University of Bergen, Post Box 7800, Bergen, 5009 Norway

**Keywords:** Oral appliance, Prediction, Obstructive sleep apnea, Endotypical traits

## Abstract

**Purpose:**

Several clinically available variables have been identified as predictors of non-response to oral appliance (OA) treatment, including endotypical traits such as severe upper airway collapsibility, unstable ventilatory control, and low arousal threshold. This study aimed to identify potential predictors of non-response to OA treatment in patients with OSA non-adherent to treatment with positive airway pressure.

**Methods:**

Patients in this study were initially treated with OAs with and without elastic bands in a crossover design. Subsequently, each patient selected their preferred treatment modality for continued therapy based on subjective preferences. The chosen OA treatment. The chosen OA treatment modality was titrated optimally based on reduction of REI. Patients not reaching > 50% reduction of REI from baseline were classified as non-responders. Statistical analyses were conducted using Student’s t-test and Pearson’s chi-squared test to assess differences in baseline variables between responders and non-responders, and logistic regression analyses were performed to investigate variables associated with not responding to OA treatment.

**Results:**

Overall, 63.2% (*n* = 36) of the patients were responders to OA treatment following titration. Smaller distance from habitual bite position to maximal retruded position (Odds ratio: 0.28, *p* = 0.016), positional OSA (Odds ratio: 0.94, *p* = 0.024) and a higher number of the endotypical OSA traits severe collapsibility, high loop gain and low arousal threshold (Odds ratio: 7.41, *p* = 0.038), were found to predict non-response to OA treatment.

**Conclusion:**

These novel findings suggest that severe upper airway collapsibility, high loop gain and low arousal threshold, identified through clinically available variables, appear to be important predictors of non-response to OA treatment, along with short distance from habitual bite position to maximal retruded position and positional OSA.

**Trial registration number:**

NCT05987618 (clinicaltrials.gov).

## Introduction

Obstructive sleep apnea (OSA) is characterized by narrowing and collapse of the upper airway during sleep, causing snoring and breathing cessations, consequently leading to hypoxia, hypercapnia and arousals during sleep [1]. It is estimated that 425 million people aged 30–69 years have moderate to severe OSA globally [2], and OSA is associated with increased risk of cardiovascular disease, daytime somnolence, and reduced quality of life [3]. Positive airway pressure (PAP) is commonly recognized as the gold standard for OSA treatment, but adherence to PAP is poor [4]. Thus, the need for alternative treatments is evident and oral appliance (OA) therapy is generally considered the second-line option [5]. Whilst adherence to OA treatment is shown to be superior compared to PAP [6], the therapeutic efficacy is highly variable [7]. Several measures have been proposed to increase success rates with OA treatment, among which better patient selection stands out as a promising approach [8].

Generally, patients with OSA have reduced passive critical closing pressure, which indicates a more collapsible upper airway. In addition to upper airway anatomy, three non-anatomical pathophysiological traits have been proposed as having substantial impact on OSA: upper airway muscle responsiveness, arousal threshold, and ventilatory control [9]. Inadequate response from upper airway dilatory muscles with increased negative pharyngeal pressure has been associated with OSA [10]. Furthermore, a low arousal threshold and unstable ventilatory control are associated with OSA due to premature arousals with narrowing of the upper airway. A hypersensitive ventilatory control system (high loop gain) contributes to inadequate responses to hypercapnia and hypoxia [10].

To individualize OSA treatment, the mentioned pathophysiological traits should guide patient categorization into endotypical subgroups to determine appropriate therapy. The gold standard for evaluating patients for pathophysiological traits involves complex and time-consuming techniques, such as analyzing flow signals from polysomnographic recordings using specialized algorithms [11] or during the “PAP-drop method” [12]. In this regard, clinical tools have been developed to assess the presence of severe collapsibility [13], low arousal threshold [14], and high loop gain [15], which enables assessment of these traits in a clinical setting.

OA treatment primarily modifies upper airway anatomy [16], and patients with OSA presenting non-anatomical pathophysiological traits, such as high loop gain and low arousal threshold, have been identified as likely non-responders to OA treatment [17]. Studies have shown that in approximately 30% of patients with OSA, non-anatomical traits play an important role in the pathogenesis [10], and that the non-anatomical traits remain unchanged post OA treatment [18]. In addition, patients having high tendency for severe collapsibility of the upper airways have been identified as likely non-responders to OA treatment [18, 19]. Therefore, identification of such patients before OA treatment, and referring them to alternative, adequate therapies, may increase the overall success rate of OA treatment.

There is a lack of studies investigating predictors for OA treatment outcomes for patients non-adherent to PAP treatment [7]. In this regard, it could be valuable to evaluate the predictive role of endotypical traits (i.e. collapsibility, arousal threshold, ventilatory control), assessed by tools using clinically available variables, amongst others. Our objective was to identify potential variables that predict non-response to OA treatment in a population with OSA non-adherent to PAP treatment. The hypothesis for the current study was that the aforementioned endotypical traits, identified using clinically available variables, are associated with non-response to OA treatment.

## Methods

### Patient selection and OA treatment protocol

Patient selection and OA treatment protocol followed the same methodology as a prior randomized crossover trial evaluating the effect of elastic bands in OA treatment, with results published in 2024 by Opsahl et al. [20].

Men and women aged 18 years and older with moderate or severe OSA who were non-adherent to PAP therapy were considered for inclusion in the study. Recruitment was conducted through the “Sleep Registry” at the Center for Sleep Medicine, Haukeland University Hospital. Exclusion criteria were mild or no OSA, inadequate dentition to support an OA, the presence of complete dentures, and the inability to read and/or speak the Norwegian language. Individuals unable to provide informed consent were also excluded.

**Fig. 1:**
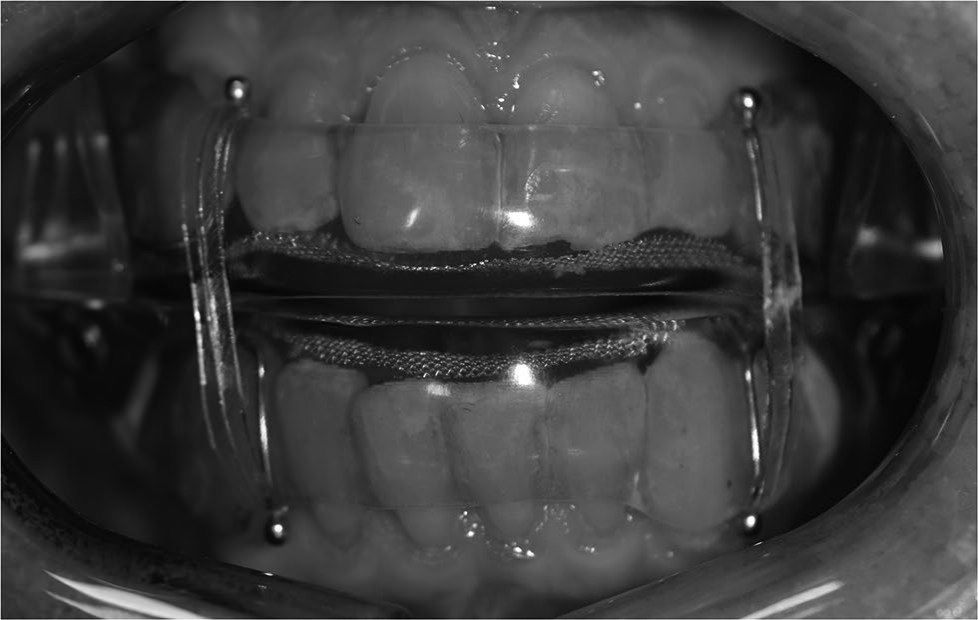
Photo of the oral appliance with elastic bands as applied in the study

Patients were treated with a custom-made OA with bibloc design (SomnoDent Fusion, SomnoMed Ltd), with and without elastic bands in randomized order (Fig. 1), with a duration of > 3 weeks for each treatment modality. Maximal protrusion was measured using the George Gauge™ bite fork, measured from habitual bite position. The overjet in the habitual bite position on the George Gauge™ bite fork replicated the intercuspal position. The sagittal distance from habitual bite position to the retruded position was also measured using the George Gauge™ bite fork. The starting position in which the OAs were fabricated was with 63% and 69% of maximal protrusion, for patients with moderate and severe OSA, respectively. These positions have been identified as optimal through stepwise, objective titration [21]. The increase of the occlusal vertical dimension was reduced to the minimal height required for the “SomnoDent^®^ Fusion” appliance (4–5 mm.) Prior to treatment start, included patients answered questionnaires regarding variables associated with sleep and OSA, and the objective effect of the treatment was investigated with home respiratory polygraphy registrations (PG) with type III devices (Nox T3^®^, Nox Medical) at the end of each treatment period, including manual scoring.

**Fig. 2:**
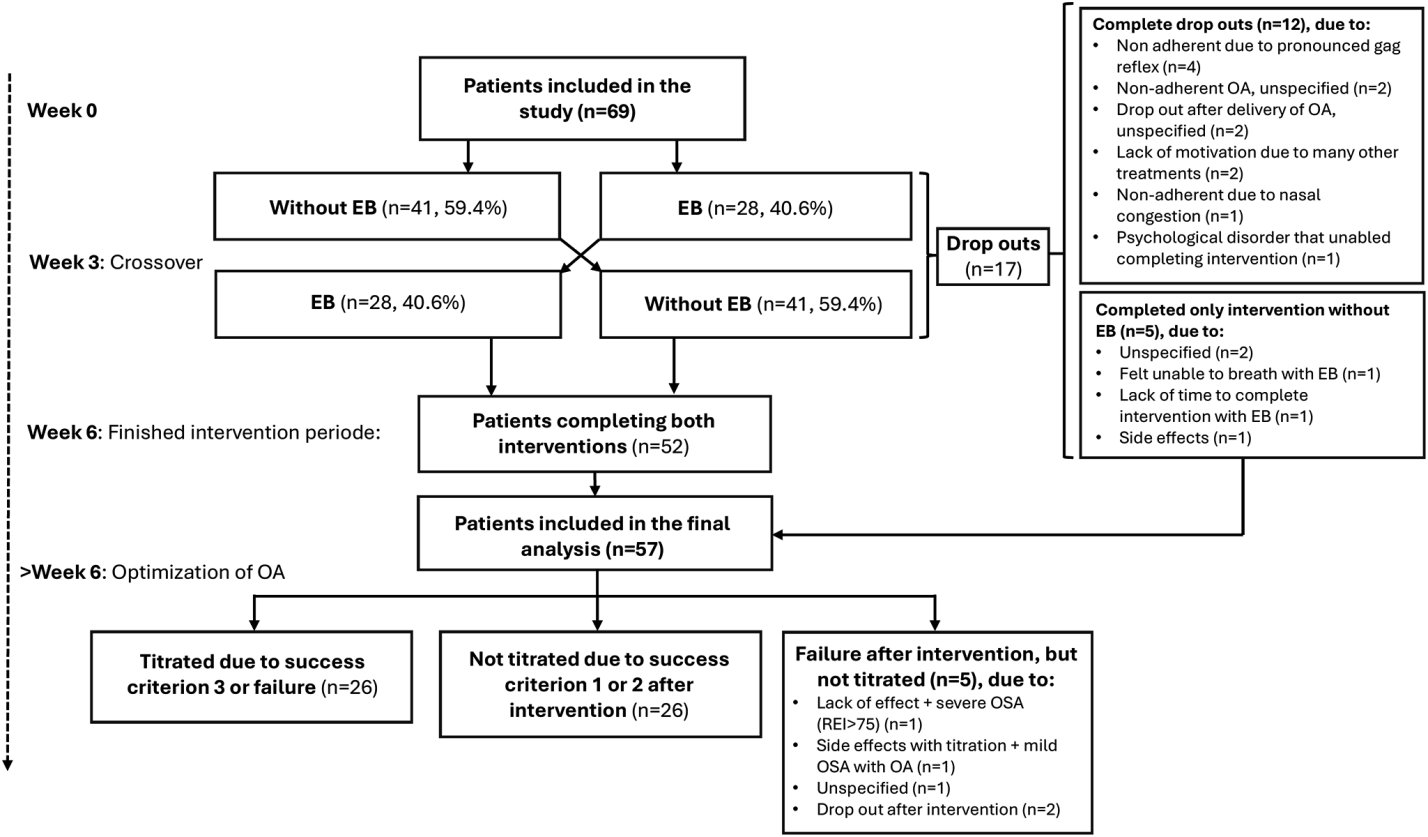
Study flow chart. *OA* Oral appliance, *EB* Elastic bands, *OSA* Obstructive sleep apnea

After completion of both treatment periods, patients were asked to choose treatment modality for further OA treatment (with or without elastic bands) based on objective effect, but also other factors deemed important by the patients, i.e. comfort and side effects. Patients who did not comply with the use of elastic bands (*n* = 5) but were able to utilize their OA without elastic bands, were included in the follow-up in this study (Fig. 2).

Success after the initial treatment, without titration of the OAs, were defined using criteria specified by Gjerde et al. [22]: REI < 5 was defined as success criterion 1, REI < 10 and > 50% reduction of REI as success criterion 2, reduction of REI > 50% as criterion 3, and reduction of REI ≤ 50% as failure. Patients achieving success criterion 1 or 2 after the initial treatment were considered successful, and not titrated further. Patients reaching success criterion 3 or being deemed as failures after the initial treatment were titrated further aiming to improve treatment effect. OAs were titrated with 1 mm increment, followed up with PG-recordings after each titration. The OAs were considered optimally titrated when reaching one or more of the following preconditions: Success criterion 1 or 2 achieved, follow-up REI did not improve after titration, or the patient felt uncomfortable with further titration of the OA.

Responders to OA treatment after titration were defined as reduction of REI > 50%, whereas non-responders were defined as reduction of REI ≤ 50%.

More detailed information regarding the study protocol, as well as the eligibility, inclusion, and exclusion criteria, is available in the publication by Opsahl et al. [20].

### Endotypical trait definitions

#### Collapsibility

It has been shown that therapeutic PAP-levels ≤ 8 cmH_2_O indicate mildly collapsible upper airway, with 89% sensitivity and 84% specificity, and with positive- and negative predictive values of 57% and 97%, respectively [13]. Thus, patients with therapeutic PAP-levels > 8 cmH_2_O are considered having severe airway collapsibility, and were scored accordingly in this study.

#### Arousal threshold

Arousal threshold was scored from available PG registrations according to a previously developed methodology [14]: Three variables were scored with one point each if fulfilled: REI < 30, nadir oxygen saturation > 82.5%, and proportion hypopneas of total respiratory events > 58.3%. Scores ≥ 2 has been shown to predict low arousal threshold with 80.4% sensitivity and 88.0% specificity [14], and patients in this study were thus scored with low arousal threshold if exhibiting ≥ 2 of these variables.

#### Ventilatory control

Ventilatory control was assessed by a validated model for clinical prediction of high and low loop gain [15]: Loop gain = 0.72 + 0.0016*REI – 0.0019*proportion hypopneas of total respiratory events. The threshold for high loop gain was set at 0.682 in this study, which was the Youden index reported in the validation study [15], with corresponding sensitivity of 0.53 (95%CI: 0.44–0.64) and specificity of 0.83 (95%CI: 0.76–0.88).

#### Number of endotypical traits per patient

The number of endotypical traits per patient was calculated by adding the number of endotypical traits severe collapsibility, low arousal threshold, high loop gain, scored per patient.

### Statistics

The statistical analyses and sample size calculation were performed with Stata version 18 (StataCorp LLC) [23].

A sample size calculation was conducted for the randomized crossover trial based on a 5% level of significance and 80% statistical power. The calculation utilized data from a pilot study with a comparable design [24], which reported treatment success rates (defined as > 50% reduction in REI) of 90% with elastic bands and 70% without elastic bands. The calculation determined that 124 participants (62 per group) would be required. Since the study followed a crossover design, this translated to a total of 62 participants. To accommodate an anticipated attrition rate of 10%, a total of 69 participants were recruited. Following the intervention phase of the randomized crossover trial, the OAs of the included patients were titrated until optimized. Data collected at the point of optimization form the basis for the analysis in this study.

To investigate differences in baseline variables between responders and non-responders to OA treatment following titration, Student’s t-test was applied to analyze continuous variables, while Pearson’s chi-squared test was used for categorical variables. The Shapiro-Wilk test was employed to assess the normality of continuous variables. For continuous variables that did not follow a normal distribution, the Wilcoxon Rank-Sum test was utilized to examine differences (Tables 1, 2, 3 and 4).

**Table 1:** Baseline demographic and anthropometric measurements in responders (> 50% reduction of respiratory event index (REI)) and non-responders (REI ≤ 50% reduction) to oral appliance treatment (*n* = 57)

Ref. category	Non-responders			Responders			*p*-value
	*n*	Mean	SD	*n*	Mean	SD	
Age, y	21	53.8	12.8	36	51.3	15.8	NS
Male sex, %	21	66.7		36	75.0		NS
Weight, kg	21	95.7	20.8	36	96.1	15.4	NS
Height, cm	21	173.4	8.1	36	177.2	7.8	*0.085*
BMI	21	31.8	6.6	36	30.7	5.3	NS
Neck circumference, cm	21	41.6	4.1	35	41.8	3.3	NS
PAP-pressure median, cm H_2_O	15	8.56	2.95	20	6.74	2.27	**0.031**
PAP-pressure 95th percentile, cm H_2_O	15	11.28	3.58	21	9.19	2.76	*0.075*
Overjet, mm	21	2.8	1.2	36	2.2	1.3	NS
Overbite, mm	21	3.5	1.6	36	2.7	2.0	NS
Maximum mouth opening, mm	21	49.7	6.1	36	52.4	6.1	NS
Maximum protrusion, mm	21	9.0	1.7	36	8.5	2.7	NS
Distance habit-retru, mm	21	2.0	1.0	36	2.6	0.9	**0.037**
Starting protrusion OA, mm	21	5.9	1.1	36	5.6	1.7	NS
Total vertical opening with OA, mm	21	9.0	1.3	33	8.1	2.1	*0.080*
Chosen treatment with EB, %	21	38.1		36	38.9		NS
Hypertension, %	20	55.0		36	38.9		NS
Diabetes, %	21	19.1		36	2.8		**0.037**
COPD, %	21	9.5		36	2.8		NS
Asthma, %	21	14.3		36	11.1		NS
Smoking, % frequency	21	28.6		36	8.3		**0.044**
Smoking, cigarettes per day	21	3.4	6.4	36	0.9	3.1	**0.042**

SD = Standard deviation, habit-retru = habitual bite position-maximal retrusion, OA = Oral appliance, Total vertical opening = Thickness of oral appliance + overbite, COPD = Chronic obstructive pulmonary disease, p-value = p-value after comparison of mean values with Student’s t-test for normally distributed continuous variables, Wilcoxen Rank-Sum test for non-normally distributed continuous variables, and Chi Squared test for categorical variables

**Table 2:** Baseline polygraphic measurements in responders (> 50% reduction of respiratory event index (REI)) and non-responders (REI ≤ 50% reduction) to oral appliance treatment (*n* = 57)

Ref. category	Non-responders			Responders			*p*-value
	*n*	Mean	SD	*n*	Mean	SD	
REI	21	32.8	17.6	36	29.4	11.0	NS
REI supine	18	38.9	17.7	33	44.0	17.3	NS
REI non-supine	15	28.5	19.4	25	18.9	13.7	*0.078*
POSA, %	13	23.1	43.9	25	72.0	45.8	**0.003**
Time supine position, %	18	38.3	26.6	30	48.8	22.5	NS
AI % of REI, %	19	40.9	27.6	30	39.5	21.0	NS
HI % of REI, %	19	58.3	28.0	30	60.4	21.1	NS
Snoring, %	17	29.0	23.9	27	27.7	17.3	NS
SaO_2_ average, %	20	91.9	2.3	36	92.9	1.2	NS
SaO_2_ nadir, %	19	76.5	7.3	36	78.5	7.4	NS
Time < 90% SaO_2_, %	21	14.9	23.6	35	7.5	8.3	NS

SD = Standard deviation, REI = Respiratory event index, POSA = Positional obstructive sleep apnea, AI = Apnea index, HI = Hypopnea index, p-value = p-value after comparison of mean values with Student’s t-test for continuous variables, Wilcoxen Rank-Sum test for non-normally distributed continuous variables, and Chi Squared test for categorical variables

**Table 3:** Baseline subjective variables in responders (> 50% reduction of respiratory event index (REI)) and non-responders (REI ≤ 50% reduction) to oral appliance treatment (*n* = 57)

Ref. category	Non-responders			Responders			*p*-value
	*n*	Mean	SD	*n*	Mean	SD	
Epworth Sleepiness Scale	21	8.0	4.4	36	7.5	4.3	NS
*Excessive daytime sleepiness*,* %*	*21*	*33.3*		*36*	*25.0*		NS
Bergen Insomnia Scale	21	16.4	9.1	36	16.9	10.1	NS
*Chronic insomnia*,* %*	*21*	*61.9*		*36*	*47.2*		NS
Anxiety, HADS	21	5.6	3.4	36	5.3	4.2	NS
*Symptoms of anxiety*,* %*	*21*	*23.8*		*36*	*25.0*		NS
Depression, HADS	21	4.5	3.5	36	4.2	3.6	*NS*
*Symptoms of depression*,* %*	*21*	*19.1*		*36*	*25.0*		*NS*
Fatigue Severity Scale, mean	21	3.7	1.4	36	3.5	1.8	*NS*
*High fatigue*,* %*	*21*	*28.6*		*36*	*25.0*		*NS*
Number of reported awakenings from sleep per night	21	2.0	1.2	34	2.9	2.1	*0.076*
Reported breathing cessations during sleep past 90 days (0 = never, 4 = always)	17	3.1	1.1	30	2.5	1.2	*0.088*

SD = Standard deviation, HADS = Hospital Anxiety and Depression Scale, p-value = p-value after comparison of mean values with Student’s t-test for continuous variables, Wilcoxen Rank-Sum test for non-normally distributed continuous variables, and Chi Squared test for categorical variables

**Table 4:** Baseline OSA endotypes in responders (> 50% reduction of respiratory event index (REI)) and non-responders (REI ≤ 50% reduction) to oral appliance treatment (*n* = 57)

Ref. category	Non-responders			Responders			*p*-value
	*n*	Freq	SD	*n*	Freq	SD	
Severe collapsibility, %	15	40.0		20	15.0		0.094
Low arousal threshold, %	18	50.0		30	43.3		NS
High loop gain, %	19	31.6		30	23.3		NS
Mean number of endotypical traits per patient	20	1.05	0.69	33	0.70	0.53	**0.041**

SD = Standard deviation,, Freq = Frequency of occurrence, The mean number of endotypical traits per patient = Calculated by adding the number of endotypical traits (severe collapsibility, low arousal threshold, high loop gain) scored per patient, divided by the number of patients per group (responders/non-responders), p-value = p-value after comparison of the categorical variables severe collapsibility, low arousal threshold and high loop gain using Chi Squared test, and the mean number of endotypical traits per patient using Student’s t-test

**Table 5:** Logistic regression model for the odds of being classified as non-responder (REI ≤ 50% reduction) after OA treatment with and without elastic bands, with “non responder” as a binary dependent variable, for the 57 patients in the study. Independent variables for the unadjusted analyses were selected based on observed differences between responders and non-responders in Tables 1–4, exhibiting p-values < 0.1 adjusted logistic regression analysis was performed with a Stepwise forward conditional method, including the independent variables exhibiting *p* < 0.1 in the unadjusted analyses. In the adjusted model, adjusted odds ratios with p-values < 0.05 were deemed statistically significant

Ref. category	Unadjusted			Adjusted		*p*-value
	OR	95% CI	*p*-value	OR	95% CI	
Height, cm	0.94	0.87–1.01	0.089			NS
PAP-pressure median, cm H_2_O	1.32	0.99–1.77	0.063	*Omitted due to missing data*		
PAP-pressure 95th percentile, cm H_2_O	1.25	0.98–1.59	0.073	*Omitted due to missing data*		
Overjet, mm	1.43	0.91–2.24	0.119			
Maximum mouth opening, mm	0.93	0.84–1.02	0.116			
Distance habit-retru, mm	0.54	0.29–0.98	**0.042**	**0.28**	**0.10–0.79**	**0.017**
Diabetes	8.24	0.85–79.44	0.068			NS
Total vertical opening with OA, mm	1.33	0.96–1.84	0.085			NS
Smoking, yes	4.40	0.97-20.00	0.055			NS
Smoking, cigarettes per day	1.13	0.99–1.29	0.066			NS
POSA	0.12	0.02–0.55	**0.007**	**0.94**	**0.01–0.74**	**0.024**
REI non-supine	1.04	0.99–1.08	0.094			*NS*
Number of reported awakenings from sleep per night	0.71	0.49–1.05	0.084			NS
Reported breathing cessations during sleep past 90 days (0 = never, 4 = always)	1.66	0.91-3.00	0.096			NS
Severe collapsibility	3.78	0.76–18.79	0.104			
Number of endotypical traits	2.81	1.00-7.86	**0.049**	**7.24**	**1.07–49.1**	**0.043**
				*Prob > chi2 = 0.0002*		
				*Pseudo R*^*2*^ *= 0.4375*		

OR = Odds ratio, CI = Confidence interval for OR, habit-retru = habitual bite position-maximal retrusion, OA = Oral appliance, Total vertical opening = Thickness of oral appliance + overbite, POSA = Positional obstructive sleep apnea, Number of endotypical traits = Adding the number of the endotypical traits severe collapsibility, low arousal threshold and high loop gain

Logistic regression analysis was performed to investigate variables associated with not responding to OA treatment after titration (reduction of REI ≤ 50%) as the dependent variable (Table 5). Independent variables for the unadjusted analyses were selected based on observed differences between responders and non-responders in Tables 1, 2, 3 and 4, exhibiting p-values < 0.10. Thereafter, an adjusted logistic regression analysis was conducted with a stepwise forward conditional method, including the selected independent variables from the unadjusted analyses. In the adjusted regression model, odds ratios with p-values < 0.05 were deemed statistically significant.

### Ethics

The study was approved by the regional ethics committee of Western Norway (protocol no: 550079 REK Vest), in addition to being approved by the health and social representative of both Haukeland University Hospital and the University of Bergen. Written informed consent was obtained by all participants before the treatment started. The study was registered at clinicaltrials.gov prior to trial start (ID: NCT05987618).

## Results

Initially, 69 patients were included in the study (19 females, 50 males). All patients were non-adherent to PAP therapy. A total of 12 patients discontinued the intervention due to various reasons, with the most frequent being a pronounced gag reflex (Fig. 2). A total of 5 patients did not tolerate OA treatment with elastic bands but were able to use their OA without elastic bands and were therefore included in the final analysis. In total, follow-up data after OA treatment optimization were available for 57 patients, comprising 16 females and 41 males. Among these patients, 34 (59.7%) had moderate OSA, while 23 (40.3%) were classified as having severe OSA. Overall, 63.2% (*n* = 36) of the patients were responders to OA treatment following titration, and 36.8% (*n* = 21) were classified as non-responders.

The variables compared between responders and non-responders are listed in Tables 1, 2, 3 and 4. Of the demographic and anthropometric variables, the mean sagittal distance from the habitual bite position to the maximal retruded position was significantly greater among the responders while therapeutic PAP-pressure, reported diabetes and number of cigarettes smoked per day were significantly lower (Table 1). Of the polygraphic variables, only frequency of positional OSA (POSA) differed significantly between the two groups, being significantly more frequent among the responders (Table 2). None of the subjective variables differed significantly (Table 3). Of the endotypical traits, severe collapsibility was found be significantly more frequent in the non-responder group, in addition to the mean number of non-favorable endotypical traits per patient, which was greater among the non-responders (Table 4).

Predictors for non-response to OA treatment after titration were (1) a small distance between habitual bite position and the maximal retruded position, (2) a high number of endotypical OSA traits present, and (3) absence of POSA (Table 5).

## Discussion

To our knowledge, this is the first study investigating variables predicting non-responders to a comprehensive treatment regime, with initial PAP treatment, followed by treatment with two different OA designs. Hence, the non-responders in this study are both non-adherent to PAP treatment and not responding to OA treatment neither with nor without elastic bands.

The variables identified as predictors of not responding to OA treatment in the adjusted model included smaller distance from habitual bite position to maximal retruded position, absence of POSA and higher number of endotypical traits assessed using clinically available variables.

A greater distance from habitual bite position to maximal retruded position measured on George Gauge™ bite fork may suggest that the mandible has a greater ability for retrusive movement during sleep. Mandibular retrusion can cause posterior displacement of the tongue, leading to mechanical narrowing of the upper airway and an increased susceptibility to collapse. Furthermore, mandibular retrusion diminishes the resting tone of the upper airway dilator muscles, necessitating greater compensatory activation of these muscles to maintain airway patency, thereby exacerbating airway instability [25, 26]. Therefore, patients with a greater distance from the habitual bite position to the maximal retruded position may have a higher risk of airway collapse during sleep. Consequently, these patients may derive greater benefit from OA treatment compared to those with a lesser tendency for mandibular retrusion, who may not experience the same advantage from limiting mandibular retrusion through OA treatment.

Preventing retrusion of the mandible with an OA in habitual bite position, without any protrusion, has shown to sufficiently treat some patients with OSA adequately in previous studies [27, 28]. Conversely, those with a smaller distance may not benefit from OA treatment to the same extent and explains its prediction for non-response. Furthermore, patients with a greater distance from habitual bite position to maximal retruded position will exhibit a larger total protrusive movement of the mandible, as the maximal protrusion was measured from the habitual bite position rather than the maximal retruded position.

The absence of POSA was also identified as a predictor of non-response to OA treatment in this study. Patients with POSA are generally more susceptible to opening of the mouth during sleep resulting in retrusion of the mandible [29]. This is also counteracted by an OA, especially an OA that does not allow mouth opening, i.e. an OA with elastic bands.

Hence, in cases where anatomical factors do not significantly contribute to the OSA pathophysiology, OA treatment is less effective. Preventing mouth opening and retrusion of the mandible are key mechanisms by which an OA achieves therapeutic effect [17].

Previous research has identified the endotypical traits severe collapsibility, high loop gain, and low arousal threshold as predictors of not responding to OA treatment [17]. Edwards et al. found that mild collapsibility and low/normal loop gain were independent predictors of AHI reduction, thereby being predictors of OA treatment efficacy [18]. Similarly, Bamagoos et al. demonstrated that OA treatment response was associated with lower loop gain, moderate collapsibility and higher arousal threshold [17]. These findings are partially in concordance with this study. Neither severe collapsibility, high loop gain nor low arousal threshold were found to be individual predictors of non-response to OA treatment in the adjusted model in our study although the numerical differences were relatively great (Table 4). Nevertheless, the number of these endotypical traits per patient were found to predict non-response, meaning that the odds of not responding to OA treatment in this study increased with the presence of any of the afore mentioned endotypical traits. Thus, it is plausible that our study may be underpowered to detect significant differences regarding these traits separately.

Many patients with OSA, particularly those being non-adherent to PAP therapy, also suffer from comorbid insomnia [30]. Despite this, patients with OSA and comorbid insomnia are frequently excluded from studies that investigate predictors of OA-treatment outcomes [17, 18]. It has been shown by Brooker et al. that patients with OSA and comorbid insomnia have lower arousal thresholds compared to patients with OSA without insomnia, but less collapsible airways and more stable ventilatory control [31]. However, Yanagimori et al. did not find a significant difference between patients with OSA with and without comorbid insomnia [32]. Consequently, patients with OSA and comorbid insomnia may exhibit different endotypical traits that are associated with response to OA treatment. The frequency of having low arousal threshold in both the responders and non-responders were comparable to other studies of previously untreated patients with OSA [14]. There was no significant difference in prevalence of chronic insomnia between responders and non-responders in our study. However, the prevalence-rates in both groups were higher compared to general prevalence rates of comorbid insomnia in patients with OSA [33].

Regarding ventilatory control, high loop gain was found in 33% of patients with OSA in a large cohort study [15]. The prevalence of high loop gain in the current study was 26.5%, which is somewhat in accordance with the findings of Brooker et al. [31], indicating that patients with OSA and comorbid insomnia exhibit more stable ventilatory control, compared to above mentioned population of patients with OSA unselected in regards of insomnia [15]. However, the lower prevalence in our study may also be due to limited sample size and estimation based on clinically available variables rather than the PSG based gold standard [15]. Further, the prevalence of severe collapsibility has previously been found to be 23% in patients with OSA [10]. Comparably, the prevalence of severe collapsibility in our study was 25.7%.

Our findings may indicate that the prevalence of low arousal threshold, high loop gain and severe collapsibility in patients with OSA non-adherent to PAP-therapy, and thus with a higher frequency of comorbid insomnia, are comparable to a previously untreated OSA-population. This suggests that patients non-adherent to PAP-therapy may be similarly predisposed to OA treatment response as a previously untreated OSA-population.

The rationale for applying the definition of a responder to OA treatment in this study (reduction of REI > 50%) was primarily based on its widespread use in evaluating success rates in comparable studies [34]. Hence, this approach facilitates a meaningful comparison of our findings with other similar studies.

This study has several limitations. First, the sample size estimation was based OA treatment success with and without elastic bands. Thus, the study may be underpowered to detect differences in endotypical traits and other predictive factors for non-response to OA treatment as the study was not originally designed or sized for this specific purpose. Secondly, we used PG registrations in our study, rather than polysomnography (PSG) registrations which were used in studies that developed the clinical tools to assess endotypical traits. PG registrations may underestimate REI in patients with milder OSA [35]. Yet, all included patients had REI > 15 at baseline, suggesting that this should not interfere significantly with the endotypical trait-assessment in this study calculated on baseline values. Nevertheless, the lower accuracy of PG registrations relative to PSG registrations constitutes a limitation of this study. Additionally, the threshold for high loop gain was set at 0.682 in this study, with sensitivity and specificity of 53% and 83% respectively, indicating that a significant fraction of patients with actual high loop gain may have been misclassified. Previous studies using gold standard methods to investigate high loop gain indicate prevalence rates of 33–36% [10, 15], higher than the prevalence of 26.5% found in our study. Third, as all included patients were non-adherent to PAP treatment, many lacked therapeutic PAP pressure data because they never were able to use PAP for an extended period. Missing therapeutic PAP pressure-data differed between the OA responder (55.6%) and the OA non-responder-group (71.4%). Further, only 38/57 patients had available position data for calculating POSA. Missing data in the adjusted logistic regression model reduces the number of observations included and may therefore impair the accuracy of the model. This should thus be taken into consideration when interpreting the outcome of the model.

## Conclusion

This study investigated predictors of not responding to OA treatment in a group of patients with OSA non-adherent to PAP-therapy and, to our knowledge, is the first to include endotypical traits identified using clinically available variables. The adjusted analysis showed that smaller distance from habitual bite position to maximal retruded position, the absence of POSA, and a greater total number of the endotypical traits were associated with not responding to OA treatment. Further research with adequate sample size estimation is needed to evaluate the predictive value of endotypical traits, identified using clinically available variables, in OA treatment.

## Data Availability

The data that support the findings of this study are available from the corresponding author, Ulrik Leidland Opsahl, upon reasonable request.
